# Ways to Improve Hospital Quality - A Health System Perspective: Comment on "Hospitals Bending the Cost Curve With Increased Quality: A Scoping Review Into Integrated Hospital Strategies"

**DOI:** 10.34172/ijhpm.2022.7422

**Published:** 2022-10-04

**Authors:** Ansgar Wuebker

**Affiliations:** ^1^RWI – Leibniz-Institute for Economic Research, Essen, Germany.; ^2^Leibniz Science Campus Ruhr, Essen, Germany.; ^3^Hochschule Harz, Wernigerode, Germany.

**Keywords:** Hospital Quality, Health System, Competition, Pro-Market Reforms, Volume-Outcome

## Abstract

Wackers and colleagues’ scoping review provides an informative and well-structured overview of hospital-based case studies focusing on integrated hospital strategies that seek to improve quality, while reducing or containing costs. Wackers et al take a hospital level perspective and evaluate facilitators and barriers to the successful implementation of those hospital strategies. I complement the hospital level perspective of Wackers et al with an analysis from a health system perspective. Regulations at the superordinate system level might influence decisions at the hospital level that are relevant for costs and quality of care. In this commentary, I discuss how interventions at the system level might affect hospital quality. The results suggest that especially competition between hospitals, pay for performance (PfP) initiatives in combination with publication of quality information, but also greater experience of hospital staff (as proxied by the volume outcome relationship) may provide impulses for improving quality of care.

 The study by Wackers and colleagues aims to identify hospitals that use management strategies to improve hospital efficiency.^1^ Using a structured scoping review, Wackers and colleagues show that hospitals use various hospital wide management strategies, such as continuos quality management, clinical pathways, Lean, Six Sigma, and value-based healthcare, to improve quality and contain costs. The authors find that hospitals that have adopted and implemented these strategies generally demonstrate better quality and lower costs. The authors also use a scoping review to examine which factors are conducive to successful adoption and implementation of the management strategies and which are rather impeding aspects. Factors to be identified relevant for implementation were categorized into eleven themes including for example the issues strategy, data information technology or skill development. Recurring factors identified as barriers for implementation of effective management strategies include lack of physician engagement, poor data collection or insufficient financial support. The authors argue that hospitals might consider the eleven conditions identified in their scoping review for successful implementation of management strategies.

 The study by Wackers and colleagues provides an informative and well-structured overview of hospital-based case studies on strategies that seek to improve quality, while reducing or containing costs. The topic is of high relevance for health policy, as the financial margins in many healthcare systems are becoming increasingly tight and successful management strategies to improve the efficiency of hospital care can make an important contribution to meeting this challenge. The study is well written and the argumentation is largely clear. The argumentation could have been sharpened only in a few sections. For example, it is not entirely clear in which direction shorter hospital stays can be interpreted, since shorter stays do not always reflect better quality, eg, in case of an inappropriate early discharge from hospital.

 Wackers and colleagues analyze management strategies initiated at the hospital level. Thereby the authors focus on hospital level determinants that promote or inhibit the adoption and implementation of management strategies. The study of Wackers and colleagues provides a relevant contribution to the literature in healthcare and hospital management. In this commentary, I want to add a health system level perspective to the hospital level perspective: Regulations at the system level might produce strong incentives for the implementation of management strategies at the hospital level.

 In this spirit, the commentary discusses “How do selected design elements of the healthcare system affect the quality of hospitals.” I focus in this commentary on the impact of pro-competetive reforms in the hospital sector, ie, reforms that introduce incentives for competition among healthcare providers.^2^ These reforms include the increased patient choice of providers, the introduction of pay for performance (PfP) shemes, the public disclosure of quality information and the opening of hospital markets for private providers. I further look at the impact of changes in the hospital market like hospital closures, accompied by the tendency to bigger and more specialized hospitals which may provide better quality, ie, the volume-outcome relationship.

## Pro-competetive Reforms

 In the last two decades many countries have introduced pro-competetive reforms in their hospital sector. The evidence on the effect of competition on hospital outcome quality is mixed and stems predomiantly from the US and UK.^2-4^ For example, Kessler and McClellan^5^ analysed consequences of hospital competition for Medicare enrolees´ heart attack care between 1985 and 1994 in the United States. They found higher mortality rates in less competitive markets, in particular in the 1990 where Health Maintenance Organization (HMO) penetration increased. Methodologically they accounted for endogeneity in patient choice and endogeneity in the competition variable. In contrast, Gowrisankaran and Town^6^ found that increasing competition for Medicare patients increased mortality rates for acute myocardial infarction (AMI) as well as pneumonia whereas increasing competition for HMO patients reduced mortality rates. The authors had a similar methodological design than Kessler and McClellan^5^ but focused only on one US state and compared the consequences of Medicare and HMO competition for different diseases (AMI and pneumonia). This mixed pattern for outcome quality also holds in more recent US and UK studies – even though the majority of well-done studies find a positive effect of competition on outcome quality.^2^

 The heterogenous findings in the literature might be, on the one hand, explained by differences in study designs, sample populations and empirical identification strategies. Moreover, it is extremely challenging to identify causal effects of competition on quality as one would need a randomized trial in the real world – ie, a randomized treatment group “competition.” On the other hand, heterogenous results might be explained by differences in the institutional setting. For instance, whether the effect of competition on quality is assessed when prices are fixed or when prices are flexible or how competition is operationalized – ie, some studies use the herfindal hirschman index whereas other studies exploit smaller elements of competition like the introduction or improvement of patient choice. Evenmore, results might depend on what type of outcome variable is assessed. Many studies use overall or indication specific mortality rates – indicators that are in the ultimative interest of the patient.^3,4^ In these studies, it is challenging to adequately account for patient selection and patient risk adjustment which might bias results. Natural experiments or instrumental variable approaches would circumvent these problems but are extreme hard to find. Other studies focus on process quality indicators (eg, the percentage of people with diabetes who had their blood sugar tested and controlled). Here, risk adjustment and risk selection are less challenging but indicators have to be in close relationship to outcome quality – the ultimative goal of the treatment – and must be measureable and easy to understand.^7^

 What can we learn from the emerging but heterogenous literature on the impact of pro-competitive reforms in the hospital sector? One take away is that results are strongly dependent on the institutional setting, such as standards with regard to hospital choice, quality reporting, quality focus or price regulation, but also on study’s methodological approach. Thus, studies that focus on particular quality dimensions in particular countries and identify the effect by using high methodological standards may contribute to a deeper understanding of incentives provided by different institutional settings and their respective effects on quality of hospital care. These studies directly relate to the hopital level quality managment initiatives described in Wackers et al^[1]^. For example, Brekke et al^2^ studied the impact of exposing hospitals in a National Health Service to non-price competition by exploiting a market based reform that increased patient choice in Norway in 2001. Using a difference-in-difference approach to mimic an experimental design, the authors found that exposure to competition reduced length of stay, AMI-mortality and all-cause mortality but increased readmissions. Comparable results are found by high-quality-studies eg, from Cooper et al,^8^ who also report decreasing length of stay and decreasing mortality rates in more competitive markets with fixed hospital prices. Likewise, theoretical work argues that quality competition in a setting under fixed prices enhances quality if prices are set above marginal costs.^4^

 There also exist a few studies that focus directly on the impact of hospital competition on more specific process quality indicators. For example, Bijlsma et al^7^ argue that process indicators are particularly important as a management tool as they are less noisy than outcome quality indicators. Thus, they can provide meaningful further information about the effect of competitive pressure on hospitals’ incentives. Their results suggest that hospitals facing more competition are better in organizing diagnostic processes; however, they have more operation cancellations at short notice and more delays of hip fracture injury operations for elderly patients. More recently Or et al^9^ tracked changes in market competition and treatment patterns in breast cancer surgery. They focused on technology adoption as a proxy of process quality and found that hospitals located in more competitive markets were more likely to apply modern treatment procedures.

 Relatedly, studies show that patients react to public disclosure of quality information and may adapt their choice of a hospital. For instance, studies reveal that while patients often choose a hospital in close distance they are also willing to drive longer to a hospital if they expect better care quality.^10^ Moreover, the disclosure of quality information affect hospital choice^11^ and increases market shares of hospitals who published better quality indicators.^12^ However, as also discussed in Bundorf et al^11^ the findings in the literature are mixed and studies point out that consumer responses to information in healthcare markets is quite small.

 As rational decision-making is often critically questioned in healthcare markets and/or there is insufficient freedom of patient choice, many healthcare systems substitute and/or complement competition by relying on PfP to reward hospitals with good quality and punish hospitals with bad quality. PfP is also intended to provide incentives for hospitals to permanently improve their quality. Empirically, results on the impact of PfP are mixed. Studies find that well-done initiatives can indeed improve quality (for an overview of the literature see, eg, Eijkenaar et al).^13^ PfP seems to work better if combined with public disclosure.^14^ However, there are also incentives to manipulate quality and select patients in order to receive higher compensation. Ultimately, the success of PfP stands or falls with the existence of valid and robust quality indicators. Outcome indicators are particularly susceptible to manipulation. In contrast, meaningful process indicators are less susceptible to manipulation and also more robust to patient selection.

 Moreover, increasing privatization of the hospital market has taken place in many health systems in recent decades. Whether privatization might improve quality and efficiency in hospital markets is a hotly debated question. On the one hand, private hospitals might be more responsive to patients needs compared to public hospitals, which might be an important source of improved quality and productivity in hospital markets (see Wübker and Wuckel, for a discussion of the literature).^15^ What is more, PfP might improve quality by adopting performance-improving innovation, particularly if combined with increased competition.^16^ On the other hand, private hospitals may be more prone to compromise patient outcomes compared to public hospitals because information assymetries might allow hospitals to cut costs at the expense of hard-to-monitor/measure quality areas.

## Hospital Closures, Specialisation and Volume Outcome

 Recent trends in hospital concentration processes may also impact quality of care. One the one hand, many studies show that higher case volume can increase quality of care through specialization and learning effects (volume outcome relationship). The closure of a hospital can thus improve care quality in the remaining hospitals where most of the patients will be treated afterwards.^17^ On the other hand, fewer hospitals in a local market may also decrease patient outcomes especially in case of an emergency. For instance, Avdic^18^ shows that emergency hospital closures decrease the probability to survive an acute myorcardial infarction.

## Concluding Remarks

 Wackers et al provide an useful systematic scoping review on hospital level faciliators and barriers for the implementation of quality enhancing and cost reducing management strategies. The authors do not address the importance of regulations at the superordinate system level, which might have a strong influence on whether and how hospital level strategies are implemented. This commentary aimed to give a small overview on how superordinate system level factors influence hospital quality. Further research might take a closer look on the interplay between regulations at the system level and hospital level initiatives to better understand how health systems could increase quality of healthcare while reducing costs.

## Ethical issues

 Not applicable.

## Competing interests

 Author declares that he has no competing interests.

## Author’s contribution

 AW is the single author of the paper.

## Endnotes

 [1] More recent studies look at competition effects on hospitals in markets with fixed prices and are specific on how they adress competition and exploit high methodolgical standards.
